# Clerical Letter

**Published:** 1863-10

**Authors:** 


### CLERICAL LETTER.

THE following communication from a former patient is well worthy of lay perusal, and is full of instruction to clergymen. It is a beacon hung out as a warning and a guide to theological students, and happy they who read it early and well. The writer has labored long and hard in the cause to which he has devoted himself, and his name is widely known in this and foreign lands.

One subject is touched, whose importance none but a physician can fully estimate, as a cause of clerical disease; it has so often forced itself upon my attention in seeing its bearing on the health and convalescence of clergymen that I have many times earnestly desired to have the ears of the whole Christian Church for an hour, in order to wake up their attention to

### ADEQUATE MINISTERIAL SUPPORT.

There are unavoidable troubles in the ministerial calling, sufficient of themselves to keep a conscientious clergyman almost always in a state of painful anxiety. I need not tell them what these troubles are, both within themselves and without; but when to all these is added the unnecessary trouble of a scanty salary, irregularly paid, seldom fully so, with wife and children at home as dear to them as life itself, whose wants must be met, and yet every source of meeting them cut off, except by the one channel, often compelled to meet these wants by credit, and then the subsequent torture to a sensitive mind of possible failure to meet the engagement, the weakening of his influence among those to whom he preaches, if "*the preacher promised to pay, and didn't do it,*" considered almost in the light of a crime, when, if the same thing were done by a man in ordinary business, it would be thought nothing of, and if done by a rich man, would not even be mentioned, for fear of giving offence—these are things hard, hard to bear, and yet it is a burden which Christian men and tender-hearted women in every section of the Church are daily imposing by the simple sin of inattention. They, in multitudes of instances, take it for granted that their minister is well cared for, and would gladly pay a fourth or a fifth of his salary themselves rather than allow them to labor under such burdens. Church-member, make it your duty this hour to see how it is with your minister.

"*Feb.* 15,

"MY DEAR SIR,—In consequence of my absence from home, the first number of your "*Journal of Health*" was not received until to-day. I had before had no intimations of its existence. Immediately upon its reception, I sat down to read it, and read it through with interest and profit. It will give me much pleasure to receive and read it regularly, from month to month, and also to embrace every suitable opportunity for recommending it to others. If it can be the means of promoting a practical acquaintance with the philosophy of living, I shall rejoice. It seems to me there is a deplorable, and almost universal ignorance on this subject. And as I look back upon the past, and consider my own deficiency in this respect, I am tempted to wish that I might live my life over again. I commenced my professional career fifteen years ago, under the most flattering circumstances. Several very eligible situations were open to me, and I had a bright prospect of extensive usefulness. But all those prospects were soon clouded, and disease seemed to put, one after another, my expectations and resolutions to flight.

"It was not, however, *wholly* owing to my ignorance of the laws of living, that I was prostrated. I am sorry to add—what a great multitude of my profession could also do—that not a little of the sad work of physical ruin was done by the *people* to whom I ministered. I had no personal enemies; but the ceaseless troubles among themselves, and *still more*, the entirely inadequate pecuniary support they gave me, and the consequent excitement and anxiety of mind, were enough, when long continued, to break down the strongest. It seems to me, my dear sir, that if you can effectually rouse the public mind, in your Journal or elsewhere, upon this most fruitful source of the numerous break-downs among ministers, you will accomplish a very great and a very important work. An extensive acquaintance with ministers throughout New England enables me to speak *what I know* on this subject. I speak here of *country* ministers; in the cities there are, so far as I know, more correct and adequate notions on the subject. We ministers open our hearts to each other about it in secret, but it is very seldom that one can be induced, especially if he *loves* his people, and earnestly desires to do them good, to disclose, even to a physician, *all* that bears upon his case as an invalid. While I fully assent to what you say of the laws of health, and know that ignorance of them is the cause of untold suffering among ministers, I also know that the treatment they receive, in the matter of worldly support, and steadfast, considerate, sympathizing *moral* aid, from those they seek to benefit and save, is doing more to cut short their usefulness, happiness, and life, than all other agencies combined. Would not your Journal be the appropriate medium of an occasional communication on this subject?

"Excuse my prolixity. When I commenced writing, I had not the slightest intention of saying anything in this strain. I designed merely to express my interest in the Journal, and to ask that a copy may be sent me.

"I am happy to say that I am still better, though tried by the inclemency and frequent changes of the weather. My little boy also continues better. I enclose one dollar for the Journal, to be directed to this place. Yours, truly."

